# The anterior cingulate cortex: an integrative hub for human socially-driven interactions

**DOI:** 10.3389/fnins.2013.00064

**Published:** 2013-05-08

**Authors:** Claudio Lavin, Camilo Melis, Ezequiel Mikulan, Carlos Gelormini, David Huepe, Agustin Ibañez

**Affiliations:** ^1^Center of Argumentation and Reasoning Studies, Universidad Diego PortalesSantiago, Chile; ^2^Laboratory of cognitive and social neuroscience, Universidad Diego PortalesSantiago, Chile; ^3^Facultad de Economía y Empresa, Centro de Neuroeconomía, Universidad Diego PortalesSantiago, Chile; ^4^Laboratory of Experimental Psychology and Neuroscience, Institute of Cognitive Neurology (INECO), Favaloro UniversityBuenos Aires, Argentina

The activity of the anterior cingulate cortex (ACC) has been related to decision-making (Gehring and Willoughby, 2002; Sanfey et al., 2003; Mulert et al., 2008), socially-driven interactions (Sanfey et al., 2003; Rigoni et al., 2010; Etkin et al., 2011), and empathy-related responses (van Veen and Carter, 2002; Gu et al., 2010; Lamm et al., 2011). We present a perspective of how to interpret the evidence of ACC involvement in these three processes, propose an ACC integrative function, and provide a methodological pathway to study decision making, empathy, and social interaction in a combined experimental approach.

**Figure 1:**
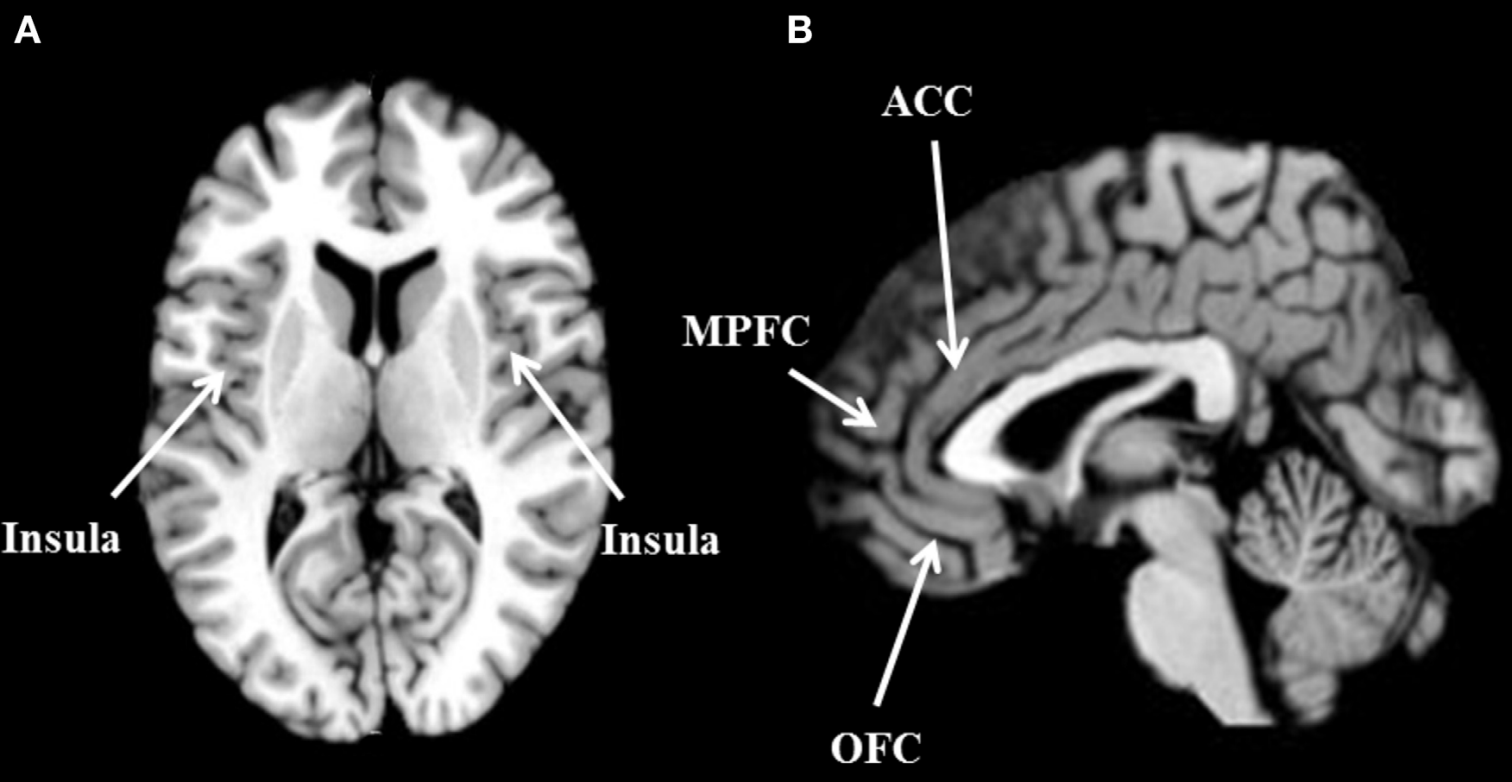
**Brain areas commonly active during empathy-related responses and decision making tasks. (A)** Axial view of the bilateral insula. **(B)** Sagittal view of the anterior cingulate cortex (ACC), medial prefrontal cortex (MPFC), and orbitofrontal cortex (OFC).

Error detection and outcome monitoring are two important decision processes related to ACC activation (Bush et al., 2000; Gehring and Willoughby, 2002; Hewig et al., 2011). Although the ACC was previously associated with basic error detection processes (Carter et al., 1998; van Veen et al., 2001), evidence from electroencephalographic (EEG) and functional magnetic resonance imaging (fMRI) during the last decade has suggested the involvement of the ACC in high-level processing (in outcome/error monitoring and action planning; Bush et al., 2000). The error-related negativity (ERN) and feedback-related negativity (FRN), two event-related potentials (ERP) that consistently follow action errors and negative outcomes, respectively (e.g., San Martin et al., 2010), are associated with activity in the ACC. The evidence of the ACC involvement in the ERN and FRN is consistent across different types of studies. In patients with ACC lesions, for instance, a robust affectation of ERN has been found (Stemmer et al., 2004; Hogan et al., 2006). Intracranial measurements confirmed ACC involvement in ERN (Brazdil et al., 2005; Jung et al., 2010), and the same evidence has been found with source localization (Dehaene et al., 1994; Holroyd et al., 1998; van Veen and Carter, 2002; Donamayor et al., 2011; Bediou et al., 2012; Ibáñez et al., 2012) and magneto-encephalography (Miltner et al., 2003). These findings are supported by fMRI studies that indicate the activation of the dorsal and rostral areas of the ACC when subjects receive feedback after losses associated with errors in decision-making tasks (Bush et al., 2002; Marsh et al., 2007). There is also animal evidence that shows specific anterior cingulate sulcus activation with respect to one's foregone rewards, and of the anterior cingulate gyrus (ACCg) with respect to self, others' or both players' rewards (Chang et al., 2013). This evidence shows that the ACC is a part of the decision-making network that involves activity in prefrontal and parietal areas related to the observation of alternatives (Platt and Glimcher, 1999; Westendorff et al., 2010), and activity in the orbitofrontal (OFC) and ventromedial prefrontal cortex related to the representation of option values (Buckley et al., 2009; Mullette-Gillman et al., 2011). There is also evidence of connections of the ACC to the insula, related to interoceptive markers of negative emotions (Ibanez et al., 2010b; Jones et al., 2011; Kunz et al., 2011; Couto et al., 2013). In addition, there is evidence that central-rostral areas of the ACC are connected to the limbic system (Etkin et al., 2011). The ACC receives inputs from these structures relative to the differences between expected and actual outcomes of a given decision, and provides outputs to coordinate dorsolateral prefrontal structures in order to organize behavioral responses (Cohen et al., 2005; Mansouri et al., 2009; Shackman et al., 2011; see Figure 1).

Furthermore, several studies show ACC activation indexing empathy-related response in pain/no-pain paradigms. The ACC is a core component of the pain network which is active when subjects receive pain stimuli and can also be activated when observing others in such situations (see Figure 1). This pain network involves activity in the bilateral anterior insula (AI), rostral ACC, brainstem, and cerebellum when observing a loved one experiencing pain, and activity in the posterior insular/secondary somatosensory cortex, the sensorimotor cortex (SI/MI), and caudal ACC when experiencing pain (Singer et al., 2004, 2006; Jackson et al., 2005, 2006; Decety and Jackson, 2006; Lamm et al., 2011). Moreover, the activation of the ACC in observational-pain paradigms is modulated by contextual information about the one observed. For instance, observing a prosocial subject receiving pain stimulation triggers empathy responses reflected in increased bilateral activity of the AI and the ACC, compared to observing an antisocial subject (Singer et al., 2006). This evidence suggests the involvement of the ACC in high-level cognitive processing when observing others and its modulation by critical contextual cues.

This high-level contextual processing of the ACC has also been studied regarding socio-affective variables within traditional decision-making paradigms. ACC is active when people observe others' action errors, but this activation is modulated by group membership of social stimuli (Newman-Norlund et al., 2009; Hein et al., 2010). ERP studies have also provided evidence in this line, showing FRN modulation associated with (1) unfairness considerations in socio-economical interactions (Boksem and De Cremer, 2010), (2) observing a friend or a stranger playing a gambling task (Ma et al., 2011), and also (3) offers made by a computer program vs. humans in ultimatum games (UG) (Fukushima and Hiraki, 2009). These neuroimaging and electrophysiological experiments suggest that ACC integrates high level information for making decisions that involve economic and social concerns. The processing in the ACC is not just related to the economic value of a given outcome, but also to the social aspects involved in the interaction. For example, the ACC activity would be differentially modulated if people, in an UG, are willing to accept unfair offers made by a computer program or by a real player (Fukushima and Hiraki, 2009). Even though the payoffs are the same, considerations about fairness/unfairness are attached to the economic interactions reflecting activity of empathy networks, theory of mind (ToM) and decision-making (Etkin et al., 2011). Although this is not conclusive of the integrative role of the ACC, the specificity of the ACC activation in decision-making paradigms when there are contextual cues, together with the role of the ACC in empathy-related responses without outcome feedback give support to this interpretation.

There is consistent evidence of the active role that the ACC plays in the processing of multimodal of context-dependent events, compared to non-contextual stimuli (Downar et al., 2001, 2002). This evidence is in line with the idea that social cognition involves the integration of flexible and context-dependent information (Chang et al., 2011; Ibanez and Manes, 2012). Taken together, these data suggest that the ACC might be a center of integration of information about others' social background that has a direct effect on economic interactions. Thus, interacting with someone from an out-group is different than interacting with someone from an in-group (Ibanez et al., 2010a) not just from a social perspective, but also in terms of how we process the economic payoffs extracted by such interactions regarding our own and others' welfare. This involves self-concern aspects of outcome processing, and empathy responses modulated by social information about others. Although we know all these processes occur to some extent in the ACC, it remains unclear which specific social cues modulate empathy in each group, and the degree to which empathy-related responses modulate cooperative behavior, outcome processing, and decision-making. In brief, most of the evidence provided focuses on just one variable (e.g., outcome monitoring or empathy) and there is no theoretical approach that has been able to integrate all variables together. Furthermore, ERP studies on the contextual cues involved in error or outcome processing tend to associate unpleasant social contexts with negative economic feedback (Boksem and De Cremer, 2010). For this reason, it is hard to evaluate the influence of contextual social cues on the processes of decision-making. Also, traditional fMRI studies, which focused on empathy, tended to put aside variables associated with outcome processing.

A further approach for studying the role of the ACC in the integration of social information, empathy and decision-making, should involve the confrontation of these factors in a single paradigm. This would allow us to observe the influence of contextual information on empathy responses, and, in turn, to evaluate whether these responses modulate the monitoring of wins and losses. For instance, fairness/unfairness considerations about others' behavior may trigger different levels of empathy-related responses depending on whether the observer profits from such behavior or not. Thus, if a given subject profits from someone else's unfair behavior, ACC activity might be affected by the economic benefit of such unfair behavior. This experimental model could explore ACC activity within conflicting situations between negative emotional states (e.g., feeling bad for observing someone being exploited or committing an error), and the positive evaluation of outcomes derived from such situations. This could show overlapping activity in the ACC, or the activation of specific areas associated with error detection, outcome processing and empathy-related responses. The same might happen when disentangling action errors from negative outcomes, as some ERP studies are doing (de Bruijn and von Rhein, 2012), where negativity associated with error detection exists even if the outcomes are positive. Such conflicts are common in real-life situations and exploring them seems essential for understanding and predicting actions within interactions under particular social settings.

The evidence summarized here supports the idea of the ACC as a center of high level contextual integration and behavior monitoring. We believe that a consistent and testable model of differential empathy-related responses using critical contextual cues (such as perceived fairness/unfairness or group identity) within a decision-making setting could provide important insights about partially overlapping ACC networks of these three cognitive domains. Real-life decision making is full of contextual cues that involve conflict between two or more alternatives at the same time (Baez et al., 2012, 2013; Ibanez and Manes, 2012). People might feel empathy for a fair player's loss but at the same time they might want to get benefits from a zero sum interaction, so there is a decision to be made in terms of which strategy weighs more in the final output. In this context, the role of the ACC would be essential for understanding how contextual information shapes our strategic decisions, and how this influences the way in which we learn from others and evaluate them in social terms.
